# Mid-term survivorship of cruciate-retaining versus posterior-stabilized total knee arthroplasty using modular mini-keel tibial implants

**DOI:** 10.1186/s13018-018-0738-9

**Published:** 2018-02-13

**Authors:** Cheng-Pang Yang, Kuo-Yao Hsu, Yu-Han Chang, Yi-Sheng Chan, Hsin-Nung Shih, Alvin Chao-Yu Chen

**Affiliations:** Bone and Joint Research Center, Department of Orthopaedic Surgery, Chang Gung Memorial Hospital-Linkou and University College of Medicine, 5th, Fu-Shin Street, Kweishan Dist, Taoyuan, 333 Taiwan, Republic of China

**Keywords:** Minimally invasive surgery (MIS), Total knee arthroplasty (TKA), Mini-keel, Modular tibial component, Radiolucency, Aseptic loosening

## Abstract

**Background:**

Reports of diverse outcomes in modular mini-keel tibial componentry for total knee arthroplasty (TKA) have raised concerns about early aseptic loosening. Cruciate-retaining (CR) prostheses, using mini-keel implants, have yet to be reported and compared to posterior-stabilizing (PS) designs.

**Methods:**

A retrospective, case-matched study of 91 consecutive TKAs (*n* = 46 CR; *n* = 45 PS prostheses), using modular mini-keel tibial componentry with a 45-mm drop down stem extension, was conducted. The Knee Society Score functional survey, radiographic analysis including alignment and periprosthetic radiolucency, TKA prosthesis longevity, and surgical complications were reported and compared between CR and PS groups.

**Results:**

The Knee Society Score at 5-year follow-up averaged 81.67 ± 11.97 and 80.12 ± 14.16 in the CR and PS groups, respectively (*p* = 0.29). The femorotibial angle averaged 5.85° ± 2.62° and 5.85° ± 3.27° valgus in the CR and PS groups, respectively (*p* = 0.60). The average tibial component angle was 0.46° ± 1.6° and 0.61° ± 1.3° varus in the CR and PS groups, respectively (*p* = 0.30); posterior inclination averaged 2.28° ± 2.36° and 1.93° ± 2.72° in the CR and PS groups, respectively (*p* = 0.51). Radiolucency was noted in 17 zones of the CR group and in 9 zones of the PS group (*p* = 0.24). Three TKAs required further surgery: one locking plate fixation for a periprosthetic tibial fracture (PS group) and two revision TKAs (one CR infection and one PS fracture).

**Conclusion:**

Modular mini-keel tibial components showed good reliability and results with both CR and PS prostheses in minimally invasive surgery TKA.

## Background

Total knee arthroplasty (TKA) is not only a cost-effective intervention for individuals with end-stage osteoarthritis of the knee but is becoming increasingly popular among individuals of working age, given the trend among working adults choosing to delay retirement [1]. As such, a less invasive approach and quick recovery have become more demanding expectations [2]. Despite yielding comparable results to conventional TKA, minimally invasive surgery (MIS) raises concerns about negative radiological outcomes and potentially higher failure rates [3–6]. Several improvements have enhanced precise instrument manufacture and modular implant design to overcome technical errors and potential misjudgments [7] in a smaller and less dissected surgical field. Modular mini-keel tibial components were developed to allow implant insertion without tibio-femoral dislocation and placement of a stem extension after positioning the tibial component, and the short-term results were encouraging [8]. However, there have been some studies reporting increasing radiolucency and the resulting higher revision rate owing to the “mini-keel” design and the additional interface with the extension stem [9, 10]. The purpose of this study was to evaluate the efficacy and longevity of the mini-keel modular tibial implant in MIS TKA and to compare the difference between cruciate-retaining (CR) and posterior-stabilized (PS) designs, based on a retrospective review of clinical and radiological findings.

## Methods

We conducted a retrospective case-matched study on MIS TKA, using modular mini-keel tibial componentry, between 2009 and 2011. Consecutive patients that underwent CR TKAs (*n* = 50) and PS TKAs (*n* = 50) were included in this study. Preoperatively, the surgical protocol and prosthesis types of all 100 patients were audited and approved by the Taiwan National Health Insurance Administration. All patients underwent unilateral TKA by the same surgeon and continued follow-up for at least 5 years. Institutional review board approval (201701327B0) was obtained to perform a review of patient records and radiographs, and informed consent was obtained from all patients. Patients with previous ipsilateral leg surgery (2 in the CR group and 4 in the PS group) or incomplete records (2 in the CR group and 1 in the PS group) were excluded. Finally, 46 CR and 45 PS TKA patients were enrolled in this study (Table 1). In the PS group, 3 out of 45 patients were scheduled to receive CR-type TKA before surgery and were subsequently changed over to a PS-type TKA intraoperatively, owing to mismatched aspect ratios with the prosthesis size [11]. There were 35 men (38.4%) and 56 women (61.6%), with an average age of 69.4 ± 7.4 years (range, 48 to 85).

**Table 1:** Demographic data

	CR group	PS group	*P* value
No. of patients	46	45	
Age	68.7 ± 7.6	69.8 ± 7.4	0.31
Sex			
Men	10 (21.7%)	15 (33.3%)	
Women	36 (78.3%)	30 (66.7%)	
Side			
Left	26 (56.5%)	20 (44.4%)	
Right	20 (43.5%)	25 (55.6%)	
Body mass index (kg/m^2^)	29.0 ± 4.7	27.5 ± 4.4	0.07
Preoperative femorotibial (°)	Varus 2.8 ± 7.2	Varus 2.7 ± 7.1	0.97

*CR* cruciate-retaining, *PS* posterior-stabilized

**Fig. 1:**
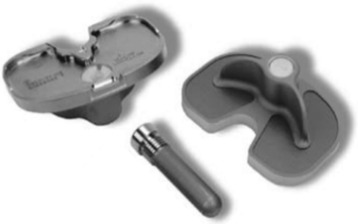
The modular mini-keel tibial implant (NexGen MIS Tibial Component; Zimmer Inc., Warsaw, IN, USA) was made of two parts: the plate with a keel underneath (mini-keel) and a modular stem

Demographic data were compared between the two groups. In the CR group, the mean age of the patients was 68.7 ± 7.6 years (range, 48 to 81). The mean body mass index was 29.0 ± 4.7 kg/m^2^ (range, 21.3 to 43.0). Preoperatively, the mean femorotibial angle was 2.8° ± 7.2° varus (range, 18.4° varus to 17.6° valgus). In the PS group, the mean patient age was 69.8 ± 7.4 years (range, 56 to 85). The mean body mass index was 27.5 ± 4.4 kg/m^2^ (range, 19.2 to 41.0). Preoperatively, the mean femorotibial angle was 2.7° ± 7.1° varus (range, 21.9° varus to 20.1° valgus). A NexGen CR-Flex Femoral Component and Fixed Bearing (CR Flex Fixed, Zimmer Inc.) was used in the CR group, and a NexGen LPS-Flex Femoral Component and Fixed Bearing (LPS Flex Fixed, Zimmer Inc.) was used in the PS group. For the tibial side, a NexGen MIS Tibial Component with a 45-mm drop down stem extension (NexGen MIS Tibial Component, Zimmer Inc.) was used in all TKAs (Fig. 1). The fully cemented technique with pressurization was used to secure the femoral and tibial components according to the manufacturer’s instructions.

**Fig. 2:**
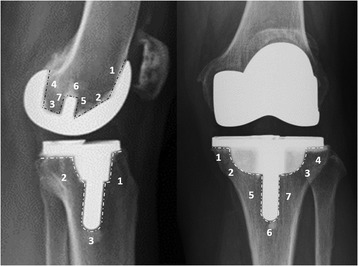
Postoperative radiographs for analysis of periprosthetic radiolucency. The tibial side was divided into 3 zones in the lateral view and 7 zones in the anteroposterior view (white dotted lines around the tibial component). The femoral side was divided into 7 zones on the lateral view (black dotted lines around the femoral component)

Clinical and radiological assessments were conducted at the last outpatient follow-up in all 91 patients. The 9 excluded patients were also available for a functional survey, either in the clinic or through telephone interview for more than 5 years afterwards, and indicated no evidence of loosening nor had they been scheduled to undergo a second surgery at the end of this study period. The clinical outcome was evaluated using the Knee Society Score [12]. Motion range of the operated knee was measured using a handheld two-arm goniometer and included total active motion, extension lag, and flexion contracture measurements. Radiological assessment included the femorotibial angle and tibial component alignment angle on a standing anteroposterior (AP) radiograph and a tibial component posterior inclination angle on a standing lateral radiograph. Regarding the status of periprosthetic radiolucency and fixation, the bone-cement-implant contact area was divided into 7 zones on the lateral view for the femoral component, 7 zones on the AP view, and 3 zones on the lateral view for the tibial component (Fig. 2). The presence of a > 2-mm-wide radiolucent line, implant subsidence, or change in the alignment was considered as a sign of radiological loosening [13].

### Statistical analysis

The demographic data, functional outcome, and radiographic assessment were compared between the CR and PS groups. Statistical analysis was calculated using SPSS version 20.0. For normally distributed data (patient age and operation time), an independent sample *t* test was used. For data that were not normally distributed (knee functional score and range of motion), the Mann–Whitney rank sum test was used. For categorical data (sex and radiographic analysis), a chi-square test was used. A *p* value of less than 0.05 was considered significant.

## Results

### Clinical and radiological results

The operation time averaged 159.9 ± 26.5 min in the CR group and 168.6 ± 20.9 min in the PS group (*p* = 0.31). Clinical outcome was evaluated on the basis of the Knee Society Score and compared between the two groups (Table 2) over an average period of 5.7 years (postoperative 5 to 8 years). The mean scores were 81.67 ± 11.97 and 80.12 ± 14.16 in the CR and PS groups, respectively, (*p* = 0.29). The average motion range of the operated knee at the final visit was 121.5 ± 13.4 and 120.9 ± 13.6 in the CR and PS groups, respectively, (*p* = 0.82).

**Table 2:** Radiological and clinical outcome

	CR group	PS group	*P* value
Operation time (min)	159.9	168.6	0.31
Knee Society Functional Score	81.67 ± 11.97	80.12 ± 14.16	0.29
Range of motion (°)	121.5 ± 13.4	120.9 ± 13.6	0.82
Femorotibial angle (°)	Valgus 5.85 ± 2.62	Valgus 5.85 ± 3.27	0.60
Tibial component angle (°)	Varus 0.46 ± 1.6	Varus 0.61 ± 1.3	0.30
Tibial component posterior inclination (°)	2.28 ± 2.36	1.93 ± 2.72	0.51
Radiolucent zones	17 (2.2%)	9 (4%)	0.24

On the basis of the latest radiological assessment, the mean postoperative femorotibial angle was 5.85° ± 2.62° valgus (range, 0.3 to 12.0) and 5.85° ± 3.27° valgus (range, 0.3 to 10.2) in the CR and PS groups, respectively, (*p* = 0.60). The mean tibial component alignment angle was 1.0° ± 1.8° varus (range, 3.9° varus to 1.9° valgus) and 1.0° ± 2.4° varus (range, 3.7° varus to 4.8° valgus) in the CR and PS groups, respectively, (*p* = 0.30). The tibial posterior inclination averaged 2.28° ± 2.36° (range, − 3.3 to 7.4) and 1.93° ± 2.72° (range, − 4.0 to 10.0) in the CR and PS groups, respectively, (*p* = 0.51) (Table 2). No evidence of femorotibial alignment change, component subsidence, or loosening was identified at follow-up.

Radiolucent lines were evaluated in each radiographic zone at the last follow-up (Table 3). In total, radiolucency was found in 17 zones (2.2%) in the CR group and in 9 zones (1.2%) in the PS group, (*p* = 0.24). There were 10 patients (11%) that showed more than one zone of radiolucency in the bone implant interface, that is: 3 CR group patients and 2 PS group patients in both the femur and the tibia; 1 PS group patient with radiolucency in the bone implant interface in the femur only; and 3 CR group patients and 1 PS group patient with radiolucency in the bone implant interface in the tibia only. In all TKAs, the radiolucency was less than 2 mm and non-progressive.

**Table 3:** Comparison of radiolucency between PS and CR groups

	Zone (case)						
PS group							
Site	1	2	3	4	5	6	7
Femur	3			1			
Tibia AP	3			3			
Tibia LAT							
CR group							
Site	1	2	3	4	5	6	7
Femur	5			2			
Tibia AP	5	1					
Tibia LAT	1		3				

### Surgical complications

There were no major intraoperative and immediate postoperative complications. Cellulitis or superficial infection was noted in 4 patients (2 in CR and 2 in PS groups) within 1 month postoperatively which responded to parenteral or oral antibiotics. One patient in the CR group experienced a periprosthetic joint infection (1.1%) and received staged surgery and a revision TKA 1 year after the primary surgery. There were two periprosthetic fractures (2.2%): a periprosthetic tibial fracture 3 months post-PS TKA, requiring locking plate fixation, and a periprosthetic femoral fracture 4 years post-PS TKA, requiring a revision TKA. The overall complication rate was 6.7% in the CR group and 8.7% in PS group. No significant difference was identified.

### Survivorship

**Fig. 3:**
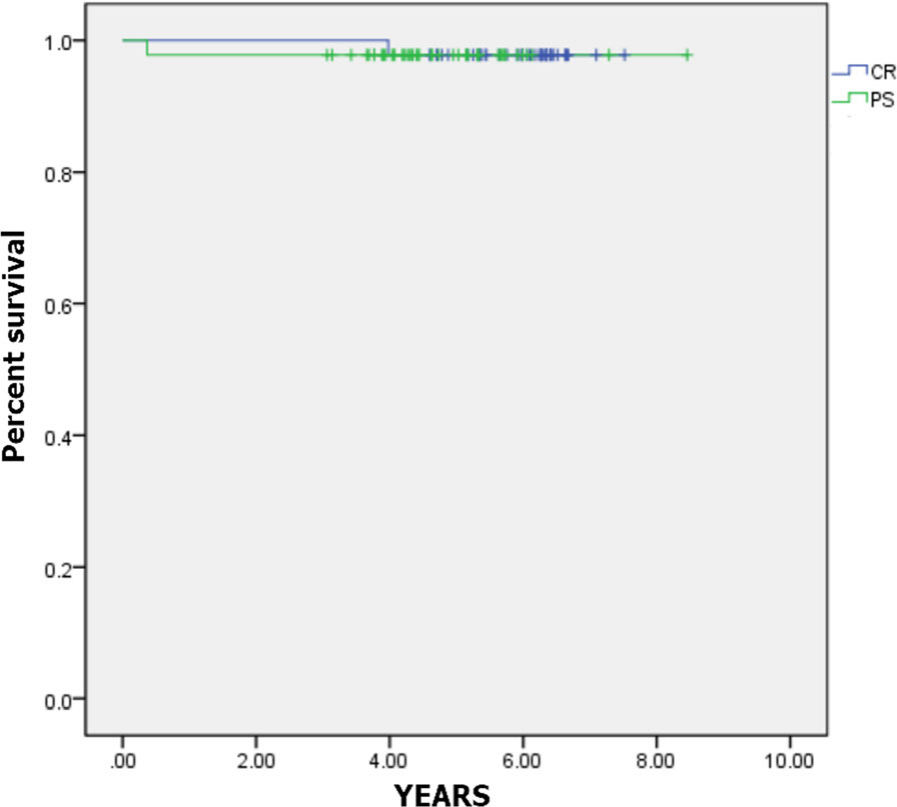
Kaplan–Meier survival analysis

The mean survival rate at 5.7 years, using Kaplan–Meier analysis, was 97.8% with a 95% confidence interval (Fig. 3). Surgical revision was required in 2 of the 91 patients that were followed for more than 5 years due to periprosthetic infection and fracture.

## Discussion

MIS TKA use has grown in the past decade owing to advances in surgical methods and instrumentation. Surgical strategies adopted in MIS TKA use are aimed at reducing the length of skin incision and damage to the underlying structures, using downsized guiding tools and cutting zigs. Potential advantages, with faster recovery of muscle strength, less blood loss, and shortened hospital stay, have been reported and have encouraged experienced surgeons to adopt these recent innovations for selective candidates [14, 15]. Modular mini-keel tibial implants were designed to facilitate insertion and manipulation of prostheses in the smaller surgical fields. Although there have been a small number of clinical studies of mini-keel tibial implants [8–10], all those studies used PS-type prostheses and showed considerable diversity in surgical outcomes. In the current study, we reported our clinical experience of modular mini-keel tibial components with CR and PS MIS TKA using functional and radiographic surveys. Both CR and PS groups showed encouraging outcomes in the 5- to 8-year follow-up period. Radiographic analysis showed the tibial component position to be 1.0° ± 1.8° varus and 1.0° ± 2.4° varus in the CR and PS groups, respectively, and demonstrated relatively high accuracy. This can be attributed to the proper recognition of surgical landmarks and the accurate application of cutting alignment guides. Furthermore, the mini-keel design allowed placing and cementing the tibial component securely without knee dislocation, while a 45-mm drop-down stem extension was additionally inserted to increase the contact surface between the tibial component and the tibia and the fixation strength [9].

Radiolucency and aseptic loosening have long been a concern in MIS TKA [16–18]. A retrospective review of 361 MIS TKAs (in 254 patients) using mini-keel tibial components reported 13.1% (22 MIS TKAs) radiolucency in the bone-implant interface [9]. The femoral and tibial components were all stable and well-fixed for longer that the 5-year follow-up period. Another clinical study of 200 MIS TKAs with mini-keel components reported 3 revisions out of 200 with a longevity of 97.4% at 3 years but did not report on the radiolucent rate [8]. While that study did not document the correlation of radiolucency, the authors emphasized the importance of cementing techniques through analyzing cement volume, distribution, and penetration from follow-up radiographs. In our study, radiolucent lines were observed in 13 of 46 patients (28%) and in 7 of 45 patients (15.6%) in the CR and PS groups, respectively. Approximately, 11% of our TKAs showed more than one zone of radiolucency in the bone implant interface. Despite the high incidence of radiolucency, all the radiolucent lines were less than 2 mm and were non-progressive; no aseptic loosening was found at the 5-year follow-up.

**Fig. 4:**
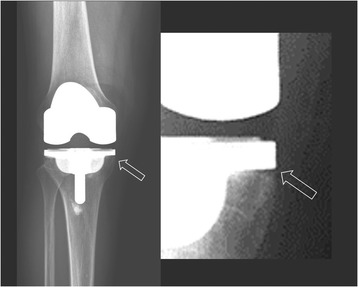
Radiolucency was more commonly seen at zone 1 of the tibial anteroposterior view owing to inadequate cement penetration (white hollow arrow)

A recently published study of 459 TKAs reported a significantly higher rate of tibial radiolucency (10.8 vs. 6.5%) and resulting aseptic loosening (5.7 vs. 1.6%) in mini-keel tibial components than in standard keel prostheses, after a mean follow-up of 5 years, and considered mini-keel design a potential risk to early failure [9]. Radiolucency was more commonly seen located around the proximal keels in both the mini-keel and standard keel groups, was slightly more common on the lateral side than on the medial side, and was more common posteriorly than anteriorly. That study differed from previous reports [10, 19] and from our study, where radiolucency was reported to be more common in the resected medial tibial surface where sclerotic subchondral bone could jeopardize cement penetration [20] (Fig. 4). In our case, none of those radiolucent lines progressed or correlated to implant loosening or periprosthetic fractures. There were 3 patients who underwent a second operation in our series. Two patients underwent revision arthroplasty: one following septic loosening and the other due to periprosthetic femoral fracture. One patient received a locking plate fixation due to periprosthetic tibial fracture, which did not go through the pre-existing medial radiolucent zone (zone 1). Since early loosening was commonly a result of malalignment and improper cementation, as is potentially anticipated in MIS TKA, all the risk factors and technical skills involved should be taken into account in preference to consideration of the mini-keel design only. In our experience, the modular tibial components permitted surgery to proceed with a reduced-tension approach because the knee was not forcefully dislocated. To insert the mini-keel tibial component accurately into the keel punch in the resected surface, the tibial component was held securely with a clamp on the anterior margin to facilitate manipulation of the implant location in the small space between the resected femoral and tibial surfaces. We applied sufficient cement for both the implant undersurface and bone resection surfaces with good pressurization and used angulated curettes and right-angled forceps with small tips to clear up all the extravasating cement, including cement debris around the retained posterior cruciate ligament in the CR group. An optimal cement amount and distribution with good pressurization is a known prerequisite for secure fixation and long-term durability of TKAs, and even more critical for tibial components with short keels.

While there were no clear and relevant differences in clinical results between CR and PS TKAs in literature reviews [21, 22], surgery with cruciate ligament sparing was generally considered to be more difficult and much more technically demanding in MIS TKA in a limited space. To our knowledge, there have not yet been any outcome reports in CR TKA with mini-keel tibial components. In the present study, both the CR and PS groups showed encouraging results with no significant difference in functional survey and radiographic analysis. The operation time was also comparable. Instead of creating more tension on distraction and dislocation of the knee joint in the flexed position during TKA surgery, the preserved cruciate ligament might help to hold the tibial end from posterior sagging in knee extension, and thus facilitate detection of the keel punch and insertion of mini-keel trial as well as the actual component correctly.

Our complication rate was 8.7% in the CR group and 6.7% in the PS group without significant difference. There were two cases of revision TKA and one case receiving fixation for periprosthetic tibial fracture. The 5-year longevity rate was 97.4%. The overall complication and prosthesis survival rates were comparable to that of standard-keel TKAs in previous studies [23, 24]. Higher incidence of periprosthetic fractures in PS type TKA warrants further survey with a larger sample size.

This study had several limitations. First, it was a retrospective study from a consecutive series using two kinds of femoral and insert prostheses. Second, despite being a case-matched study, some cases were converted to the PS-type prosthesis intraoperatively. The decision for conversion was determined through the planning and experience of a single surgeon. Third, there was a relatively small sample size in each group. Since the government has requested preoperative registration and audits the intraoperative conversion rate, these regulatory requirements limited our ability to extend the study to increase patient numbers. Finally, the clinical and radiographic results were not compared to those of other MIS TKAs using the standard-keel prostheses at our institute. However, this step was not necessary to fulfil the main objectives of the present study.

## Conclusion

The data of the present study demonstrated encouraging outcomes and good reliability of modular mini-keel tibial implants with MIS TKA in both CR and PS groups. Periprosthetic radiolucency evaluated at 5 to 8 years postoperatively indicated neither progression nor a tendency for aseptic loosening. Further extended investigation is essential to determine the longevity and definite advantages following this surgery.

## Abbreviations

AP: Anteroposterior
CR: Cruciate-retaining
MIS: Minimally invasive surgery
PS: Posterior-stabilizing
TKA: Total knee arthroplasty
